# Design and Validation of Multichannel Wireless Wearable SEMG System for Real-Time Training Performance Monitoring

**DOI:** 10.1155/2019/4580645

**Published:** 2019-09-09

**Authors:** Serkan Örücü, Murat Selek

**Affiliations:** ^1^Ermenek Vocational School, Karamanoğlu Mehmetbey University, Karaman 70400, Turkey; ^2^Vocational School of Technical Sciences, Konya Technical University, Konya 42130, Turkey

## Abstract

Monitoring of training performance and physical activity has become indispensable these days for athletes. Wireless technologies have started to be widely used in the monitoring of muscle activation, in the sport performance of athletes, and in the examination of training efficiency. The monitorability of performance simultaneously in the process of training is especially a necessity for athletes at the beginner level to carry out healthy training in sports like weightlifting and bodybuilding. For this purpose, a new system consisting of 4 channel wireless wearable SEMG circuit and analysis software has been proposed to detect dynamic muscle contractions and to be used in real-time training performance monitoring and analysis. The analysis software, the Haar wavelet filter with threshold cutting, can provide performance analysis by using the methods of moving RMS and %MVC. The validity of the data obtained from the system was investigated and compared with a biomedical system. In this comparison, 90.95% ± 3.35 for left biceps brachii (BB) and 90.75% ± 3.75 for right BB were obtained. The output of the power and %MVC analysis of the system was tested during the training of the participants at the gym, and the training efficiency was measured as 96.87% ± 2.74.

## 1. Introduction

In recent years, the monitoring of athlete performance has become indispensable for the health of athletes. Wireless technologies have started to be widely used in order to obtain data for the purpose of examining training efficiency in the monitoring of muscle activation and sport performance of athletes [1, 2]. It is possible to collect information about athlete performance and rehabilitation, about preventing muscle fatigue or injuries through posttraining analysis of SEMG signal obtained during the training [3–5]. Recording of SEMG signals in related muscles during training can be extremely useful in increasing performance and preventing disabilities [6].

Traits of SEMG signals obtained during training (frequency, severity, etc.) change depending on the muscle group measured and the severity of contraction [7–9]. In these measurements, surface-type electrodes are used to determine and examine the activity of muscles during contraction and relaxation of muscles. When academic studies related to this subject are analysed, there are some wearable biometric systems developed for the purpose of the monitoring of performance during training. Some of these systems are intended for recording parameters like heart rate, respiration, location, and velocity or for estimating the levels of muscle fatigue [10–13]. Some of them have been produced for the measurement of the SEMG signals in laboratorial environment [14]. Another proportion of them has been designed for the purpose of perceiving dynamic muscle contraction during isolated training through the SEMG [15]. The last proportion has carried out low-cost experimental SEMG systems and matched the key features of the system with the existing systems [16–18].

The most reliable method used in the adequacy and examination of muscle activation in physiological studies is the amplitude analysis carried out on SEMG signals, known as MVC (maximum voluntary contraction) normalization [19]. Data with MVC normalization enable understanding of what capacity the muscle works, how effective level muscles have reached through training and how much effort a training requires from an athlete [20].

The simultaneous monitorability of athlete performance during the process of training is a must for athletes at the beginner level to being able to carry out healthy training in sports like weightlifting and bodybuilding [21, 22]. This feature enables performance evaluation to be carried out momentarily during the time when there is no trainer or until the motor skills of the athlete concerning movement develop enough. A SEMG system, to be used during the training for this purpose [7, 23–25] has to

- Be able to provide the required SEMG data necessary for monitoring training efficiency in performance analysis
- Be able to filter the noise of movement during isotonic exercises and noise and distortions in SEMG signals appearing as a result of other factors
- Its procedures like calibration, etc., have to continue for a short time
- The data obtained have to be at a close accuracy to biomedical systems
- Has to be simultaneously usable in a training environment

For use in the industrial field, various systems are available for SEMG data collection and processing. To investigate these, WB-EMG [26], BiometricsDatalog [27], Myo Armband [28], DelsysTrignio [29], BITalino [30], Mbody3 [31], Mpower [32], MyoTrac [33], MyoWare [34], Shimmer [35], and hospital [36] are such systems. The systems specified in [26], [27], and [29] and the systems which we measure in hospital [36] are not wearable during training. The system specified in [44] is wearable and supports wireless transmission but its production is stopped. In terms of the electrodes used, and CMRR, there is no difference in all of these products and they comply with the SENIAM criteria. The systems [26], [27], and [30] do not have noise and data processing filters, and the systems in [28] and [29] use a Notch filter and a band-stop filter with narrow-bandwidth in hardware. The system in [26, 34] is designed for single-channel use but does not support multichannel monitoring. The systems in [31–34] are wearable and do not include contraction detection and simultaneous MVC analysis although they can monitor multiple muscle groups. A summary of these comparisons is presented in Table 1.

When the table is analysed, it is seen that all of these systems can simultaneously observe biopotential changes in muscle or muscle groups monitored during training, but none of them include real-time MVC normalization and contraction detection procedures for performance analysis during training.

That these features can be monitored simultaneously during the training process may be useful especially for beginner athletes to perform a healthy training in sports like weightlifting and bodybuilding, for the performance evaluation of the athlete until the motor skills of the movement are improved and at necessary moments in preventing the injury process by intervening in training.

Based on these elements, a new wireless wearable SEMG data collection system has been introduced which enables performance monitoring and analysis at training time with its real-time MVC normalization and contraction detection processes. The SEMG circuit used in our system is designed by us to be used in future studies and to be developed according to our needs.

In the presented system, digital filtering is also used in addition to hardware filtering in SEMG circuit. These numerical filters are Haar wavelet filters with Threshold cutting based on (TCHW) and linear Kalman [37, 38]. Each numerical filtering method is tested together with hardware filtering. Results obtained from here will be determinative in deciding the filtering structure that can be used in future stages of the system design. Subsequently, filtered data are processed through moving RMS method containing the methods of moving average (MA) and root mean square (RMS), scaled through MVC normalization, and a training support system that can carry out real-time performance analysis and monitoring.

## 2. Materials and Methods

### 2.1. Isotonic Contraction

Isotonic contraction encompasses exercises where muscle tendons get shortened to generate movement. Any kind of movement, ranging from weightlifting to rowing and running, is in this category [39]. In sport, an isotonic exercise is a training where the most amount of strength is exerted on a particular muscle or muscle group to increase that muscle mass or performance in general. Due to the fact that human activity and athletic performance necessitate these kinds of movement, isotonic exercises form the basis of a lot of training protocols [40]. It is possible to observe pathological changes or efficiency obtained from the training through an examination of SEMG signals generated in muscles during these exercises [41].

### 2.2. SEMG Circuit Design

**Figure 1:**
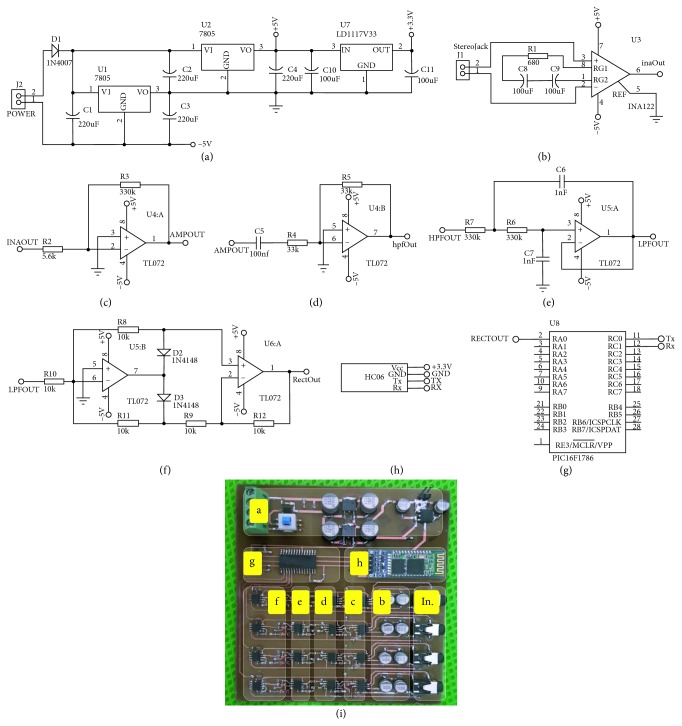
Block diagram and mounted state of the SEMG circuit. (a) Regulator circuit. (b) Instrumentation amplifier. (c) Inverting amplifier. (d) 1^st^-order HPF. (e) 2^nd^-order Sallen–Key LPF. (f) Full-wave rectifier. (g) PIC 16F1786. (h) Bluetooth module. (i) Mounted state of the SEMG circuit.

The SEMG circuit design details are given below. The circuit consisting of 4 channels could monitor the biopotential change of 4 different muscle groups at the same time. So, it is possible to monitor biopotential changes occurring in muscles in symmetrical movements that affect multiple muscle groups (e.g., the Bench Press movement affects pectoralis major and triceps muscles). The circuit has in each channel, respectively, one instrumentation amplifier, a inverting amplifier, a low-pass filter, a high-pass filter, and a full-wave rectifier. The circuit has a diode for input protection, a pointer indicating that the circuit is working, and a start-up button. During working, the LD1117 regulator was used for the Bluetooth feed and the 7805 regulator for the +5 volt and −5 volt op-amp feed (Figure 1(a)). The SEMG signals we want to process are MUAP signals whose amplitude is between 0 and 1.5 mVolt (RMS). To process this electrical signal, it must firstly be amplified. In the system, this amplification is done by increasing the difference between the two electrodes in bipolar mode. While the obtained common signal is amplified in this mode, the background noise is also suppressed. Two of the probes activated from each channel are connected to the circuit's soil, like the reference probe [42] which is placed in a more electrically remote area (preferably a neutral or close to the bone region) while going to the amplifier and filter circuits over INA 128P, which operates in a single differential mode. In the first step, amplification was performed by using the INA 128P differential amplifier (Figure 1(b)).

As stated in [43], the reason why we use INA 128P is that the amplitude of the SEMG signal is low and that the amplifier to be used due to other factors like noise must have a high input impedance and a high common mode rejection rate (CMRR > 95 dB). This amplifier has the required features with CMRR >120 dB and 10 GΩ input impedance. When we set the gain value for the 60 Hz input signal to *G* = 74.52 using INA 128P in our system, approximately 108 dB CMRR was obtained as stated in the technical document in [44]. The reason for selecting a 60 Hz input signal in the system design is that the SEMG signal is dominant in the range of 50 Hz to 150 Hz. To obtain a processable signal amplitude in the second stage, TL072 was used as shown in Figure 1(c) as an active inverting amplifier. At this stage was the amplifier gain approximately *G* = 59 and the CMRR approximately 100 dB by using the 60 Hz input signal as stated in [45].

In SEMG applications, analogue (hardware) and digital (software) filters are used to remove unwanted component noises and process the necessary parts in the SEMG signal [46]. Analogue filters remove anything above or below a selected cut frequency, while digital filters make this process more precise as they can be programmed [47]. This certainty is due to the fact that the features of digital filters can be changed depending on the input signal parameters [48]. In these applications, analogue filters are used to eliminate noise from the signal in signal amplification and processing circuits, to provide noise immunity, and to obtain the necessary parts of the frequency band [49]. On the contrary, digital filters are used to filter signal residues named artifact after motion and to analyse SEMG signal (feature extraction, time-frequency analysis, contraction detection, performance analysis, etc.) [41, 50].

In the circuit, analogue filtering is performed by low- and high-pass filters. Ideal SEMG signals are observed between 50 Hz and 500 Hz and should be filtered from frequency components outside this range [51]. For this, the signal from the output of the instrumentation amplifier is first filtered so that the gain is 1 in the high-pass filter (HPF) using TL072 with a cutoff frequency of about 48 Hz (Figure 1(d)). The components of the EMG signal above 500 Hz are filtered through a 2nd order Sallen–Key low-pass filter (LPF) using TL072. Through this section, resistance and capacitor values are designed so that the cutoff frequency is approximately 482 Hz, the quality factor is 0.5, and the gain is 1 (Figure 1(e)). The reason we prefer the Sallen–Key topology we use in the circuit is that this filter has the ability to produce a quadratic low-pass reaction with better selectivity (higher pole) and various possible approaches (Butterworth, Chebyshev, Thomson-Bessel, etc.) [43, 47, 49]. This will help us in our future work.

Then, the whole SEMG signal was moved to the positive level using the full-wave rectifier (Figure 1(f)). With this process, it is possible to analyse the low-frequency oscillations by overcoming the high-pass nature of the SEMG signal [52]. Thus, it is aimed to use the circuit except for the training efficiency, also in the fields of prosthesis control and ergonomics.

The Pic16F1786 microcontroller with connected full-wave rectifier outputs contains 11 12 bit A/D (Analogue/Digital) converters. The data obtained from the rectifier of each channel in the circuit are connected, respectively, to the RA0-RA3 inputs of this controller. This microcontroller performs the A/D conversion in 20 ms time intervals through the program we write. The converted channel data are turned into a string, and this sends data from the RC0 output to the Bluetooth module (Figure 1(g)). The transmitted data have a resolution of 2.4 *μ*V in each step. Data sent at 4800 bps speed via the HC-06 Bluetooth module (Figure 1(h)) are received and processed by the data collection program written in the C# language. The digitalized SEMG data in the data collection program are processed through digital filters. The PCB (printed circuit board) of the circuit is designed to be 10 cm × 10 cm in size, and as stated in [53], the PCB tracks are intended to be exposed to as little noise as possible. The mounted state of the circuit shown in Figure 1(i) is boxed and placed inside a wearable belt. The necessary energy for the operation of the circuit was obtained from 1000 mAh lithium batteries. It is intended to minimize power line interference (PLI) without the need for any insulation, as stated in [54] using battery in the system.

### 2.3. Participants and Setup

Five males and two females voluntarily participated in our study and have at least 2 years of experience in strength training. The information of the participants is shown in Table 2.

**Figure 2:**
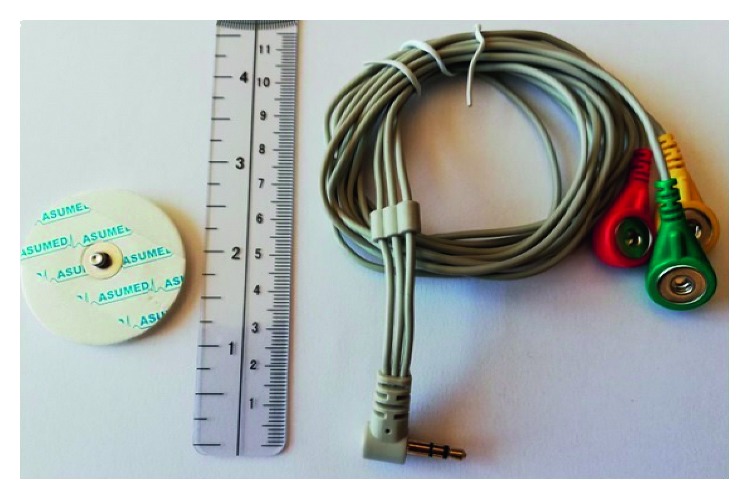
Example view of electrodes and shielded cables.

The participants were informed about the content of our study, and a signed consent form was obtained from all of them. All exercises and measurements were made under the supervision of a specialized trainer. As described in the recommendations of the European initiative known as SENIAM (surface electromyography for noninvasive muscle evaluation of muscles) by selecting 10 mm diameter electrodes shown in Figure 2 for SEMG, the bipolar configuration is located 1–2 cm away from the centre of the muscle and the reference electrode is placed in a region that is electrically neutral according to the action [51]. The connection between the electrodes and the circuit channels is provided by using armoured cables which have 3.5 mm ends, 3 colour code (red, green, and blue) and labelled contacts (L, F, and R), as shown in Figure 2.

Our experiments consist of 3 parts. In the first part, 8 repetitions and 1 set of alternate dumbbell curl (ADBC) training was performed using a maximum load of 60–70%. In this section, firstly, it is investigated whether the analogue filter data obtained from the circuit in the training reflect the biopotential activity changes that occur during the training. In the sequel, the analogue filter data obtained from the circuit are processed by means of Kalman and threshold cut Haar wavelet filter (TCHW) to eliminate noise sources and to investigate the perceptibility of the isotonic contractions.

In the second part, the accuracy of the developed system was compared with the biomedical system (Viking on Nicolet EDX) used in Karaman State Hospital (See Table 1). In this comparison, the RMS values obtained from both systems were used.

In the third part, the availability of moving RMS and %MVC values as the screen output of the system was investigated in terms of performance feedback. For this purpose, first, the moving RMS values obtained by asking users to perform a second ADBC (8 repetitions 1 set) training were recorded. In addition, a %MCV measurement was made by asking all users in the training environment to lift 5 kg dumbbell and maximum weight (Men 17.5 kg, 20 kg, and 25 kg dumbbell; women 12.5 kg and 15 kg dumbbell) they can.

### 2.4. Kalman and TCHW Filters

Kalman filter is used to estimate the system status from input and output information with the previous information of a model in a dynamic system indicated by the state-space model [55, 56]. When the system is modelled, it was aimed to minimize the distortions in data by estimating the *k* parameter specified by *x* in SEMG data array at a particular time as *X*_*k*_:

(1) $$\begin{array}{c} {\hat{X}}_{k}=K_{k}\cdot Z_{k}+\left(1-\right)K_{k}\cdot {\hat{X}}_{k-1}. \end{array}$$

Here, *Z*_*k*_ expresses the measuring data wanted to be absolutized, *K*_*k*_ the Kalman gain and *X*_*k*−1_ the measuring data belonging to the previous stage. If the system is modelled through this information, a model consisting of calculation (2) and update (3) is obtained.

(2) $$\begin{array}{c} x_{k}=Ax_{k-1}+Bu_{k}+w_{k-1}, \end{array}$$

(3) $$\begin{array}{c} z_{k}=Hx_{k}+v_{k}. \end{array}$$

In (2), any *x*_*k*_ is expressed as a linear combination of the next control signal *k* of its previous value and the noise of the process. In (3), any measurement value making certain of the accuracy of which we are not sure is accepted to be a linear combination of the signal value and the noise of the measurement.

In HW, the main wavelet acts as the wavelet transform but is scaled and shifted during this procedure of wavelet transform [35]. Scaling corresponds to the widening and constriction of the signal (*f*(*t*)) and the shift to the wave shift (*τ*) in the timescale axis (*t*) in the following equation [57, 58]:

(4) $$\begin{array}{c} F\left(\omega ,\tau \right)=\int f\left(t\right)w\left(t-\tau \right)e^{-j\omega t}dt. \end{array}$$

HW is a wavelet-based, scaled, “square-shaped” array of functions. *ψ*(*t*), the main function of HW (5), and also *φ*(*t*), a scaling function (6), are defined in *t* time interval given as follows:

(5) $$\begin{array}{c} \psi \left(t\right)=\left\{\begin{array}{cc} 1, & 0\leq t\leq \frac{1}{2},\\ -1, & \frac{1}{2}<t\leq 1,\\ 0, & \text{otherwise}, \end{array}\right. \end{array}$$

(6) $$\begin{array}{c} \varphi \left(t\right)=\left\{\begin{array}{cc} 1, & 0\leq t\leq \frac{1}{2},\\ 0, & \text{otherwise}, \end{array}\right. \end{array}$$

The Haar function *ψ*_*n,k*_ is defined as shown in

(7) $$\begin{array}{c} \psi_{n,k}\left(t\right)=2^{n/2}\psi \left(2^{n}t-k\right),\;t\in ℜ. \end{array}$$

Since the SEMG signals are user-based, SEMG signals between isotonic muscle contractions may vary according to the individual. In the method we use with HW, the individual waits for approximately 2–4 seconds with the weight in his hand before starting training and in the meantime, the procedure of threshold cutting in the system can be carried out. The threshold cutting is based on the calculation of the average value (8), the standard deviation (9), and the signal slope (10):

(8) $$\begin{array}{c} A=\frac{1}{n}*\overset{n}{\underset{i=1}{\sum }}x_{i}, \end{array}$$

(9) $$\begin{array}{c} \sigma =\sqrt{\frac{1}{N}\overset{N}{\underset{i=1}{\sum }}{\left(x_{i}-\mu \right)}^{2}}, \end{array}$$

(10) $$\begin{array}{c} s=\frac{\sum \left(x-\bar{x}\right)\left(y-\bar{y}\right)}{\sum {\left(x-\bar{x}\right)}^{2}}. \end{array}$$

Here, *x*_*i*_ is the value added to the average, *μ* is the average value and *N* is the number of the total value. After the values of the average, standard deviation and slope are calculated and all SEMG signals complying with this condition are equalled to zero. Thus, the signals between the voluntary contractions can be eliminated.

### 2.5. RMS, MA, and %MVC

After the SEMG signal is captured, the commonly used RMS or MA values are analysed by using [59]. In RMS analysis, the SEMG signal is subjected to a set of mathematical operations designed to measure the power of change. Thus, the intensity and duration of events like muscle contractions can be investigated. Therefore, the RMS value is a parameter chosen during contraction and reflects the level of physiological activity in the body. Mathematically, the RMS value of a continuous-time waveform is the square root of a function defining the continuous waveform shown in  *f* (*t*) in the following, defined in the range *T*_1_ ≤ *t* ≤ *T*_2_:

(11) $$\begin{array}{c} f_{\text{rms}}=\sqrt{\frac{1}{T_{2}-T_{1}}\int_{T_{1}}^{T_{2}}{\left[f\left(t\right)\right]}^{2}dt}, \end{array}$$

(12) $$\begin{array}{c} f_{\text{rms}}=\lim_{T⟶\infty }\sqrt{\frac{1}{T}{\int_{0}^{T}\left[f\left(t\right)\right]}^{2}dt}. \end{array}$$

Another method we use as MA is the technique of analysing changes in a data set to estimate long-term trends. For a given N time window, if the values *s*_1_, *s*_2_, *s*_3_,…, *s*_*n*_ corresponding to this time interval of the S variable shown in the times *t*_1_, *t*_2_, *t*_3_,…, *t*_*n*_ are known, the MA window size is defined as *N* = 2*k* + 1 and processed as specified in

(13) $$\begin{array}{c} \text{MA}=\frac{1}{N}\overset{+k}{\underset{j=-k}{\sum }}s_{i-j}. \end{array}$$

Thus, changes in the time window given at the *j* moment are obtained by averaging the time series of the *k* time in the *j* moment. Instead of using the abovementioned RMS and MA methods separately, the moving RMS method was used in our system by calculating the RMS value in a moving window, which is a combination of these methods. In this method, the operation can be performed at any *t* time interval of the moving window; therefore, it acts as a filter in a certain time interval, as shown in (14). In this way, the processing of the data obtained according to the variable speed of the replays in the training sets gets easier. In this equation, *n* refers to the length of the window, while *x*(*k*) refers to the data within the window:

(14) $$\begin{array}{c} x_{\text{RMS}}\left[i\right]={\left(\frac{1}{n_{0}}\overset{i}{\underset{j=\left(i-N+1\right)}{\sum }}x^{2}\left[k\right]\right)}^{1/2}. \end{array}$$

So, it can be measured how much power is obtained from the muscle through the moving RMS value.

The MVC (maximum voluntary contraction-maximum amplitude of the signal) normalization is widely used in SEMG signals as an amplitude analysis technique. The results are shown as a percentage (%MVC) of the MVC value that can be used to create a common background when comparing data between subjects [60, 61]. SEMG signals depend on the user and have a structure that can cause records to change even when measured from the same position with the same motion. Therefore, MVC normalization is used to eliminate this difference and to enable data comparison between subjects [61]. MVC expresses the highest value obtained in a repeat during this measurement to normalize SEMG signals obtained for a specific purpose, while SMVC (submaximal voluntary contraction) refers to the voluntarily recorded SEMG data. %MVC corresponds to the multiplication of the normalized value of according to SMVC's MVC with 100 [62, 63]:

(15) $$\begin{array}{c} \%\text{MVC}=\left(\frac{\text{SMVC}}{\text{MVC}}\right)*100. \end{array}$$

Thus, it can be scaled how much power is obtained from the muscle or muscle groups investigated in repetitions in each set of training.

### 2.6. Proposed System

**Figure 3:**
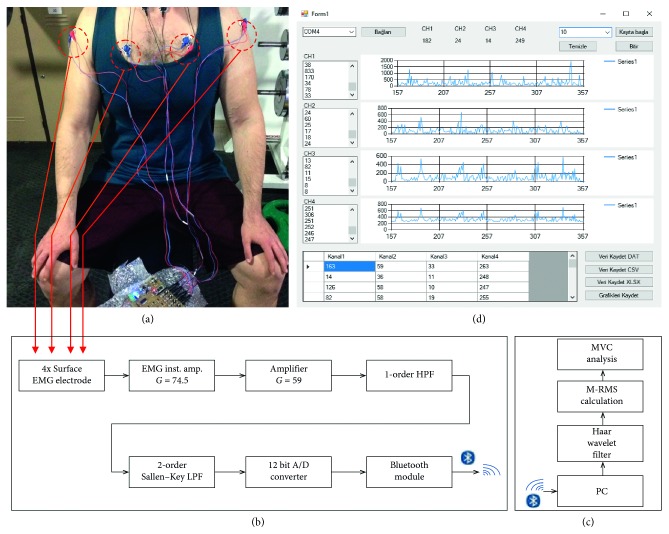
Overview of the system. (a) Connecting electrodes before training (Photoshoot by Orucu). (b) Block diagram of the SEMG circuit. (c) Block diagram of the analysis software. (d) User interface of the analysis software.

Our system has the ability to follow the biopotential changes of four different superficial muscle groups at the same time. The reason why the system is designed with 4 channels is that most movements used in bodybuilding and weight training activate at least 1 or 3 muscle groups at the same time. The system takes the biopotential signals of the muscles that are activated during training through surface electrodes (Figure 3(a)), and then, first it amplifies them in the instrumentation and amplifier parts in the SEMG circuit, after it filters them with the 1^st^-degree high pass and 2^nd^-degree Sallen–Key low-pass filters. These analogue-filtered signals are sent to the computer via Bluetooth after a 12 bit analogue-to-digital conversion (Figure 3(b)). By the software we developed in C# language, all SEMG channel data received by the computer are digitally filtered and then they calculated the moving RMS values in time windows that vary according to the training speed (Figure 3(c)). After this process, the SMVC value of each repetition in each set of the training is processed according to the previously saved MVC values. Then, %MVC values are displayed on the screen in separate graphs according to the channels from which the data are taken. Finally, they are saved to the database in “.csv,” “.dat,” and “.xlsx” formats (Figure 3(d)).

## 3. Results and Discussion

### 3.1. Analogue + Digital Filtered Data from the System

**Figure 4:**
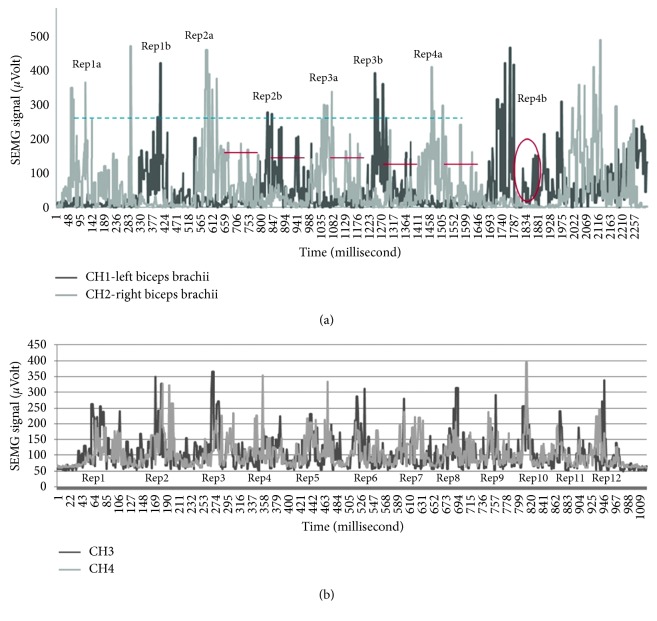
Sample analogue filtered data obtained from the SEMG circuit during training: (a) Sample results of participant number two, (b) sample results of participant number six.

The analogue-filtered data of the first 4 repetitions of ADBC training performed by participant number two is shown in Figure 4(a), marked as 4(a) and 4(b) for each repetition.

The left BB (LBB-Left Biceps Brachii) data are obtained from CH1 (first channel of the SEMG circuit), and the right BB (RBB-Right Biceps Brachii) data are obtained from CH2 (the second channel of the SEMG circuit). From the data obtained, some decrease in Rep2b, Rep3a, Rep3b, and Rep4a (between 100 and 200 *μ*V) and a data change during pushing the weight down (relaxation period of the muscle) in Rep 4b were observed. As we consulted with the professor of Physical Education and Sports Teaching (Karamanoğlu Mehmetbey University), he stated that the fall was caused by the distortion of movement. According to the consultant professor, this change appeared to have been caused by the prolongation of the activation period of the muscle as a result of pushing the weight down more slowly as specified in [64, 65].

**Figure 5:**
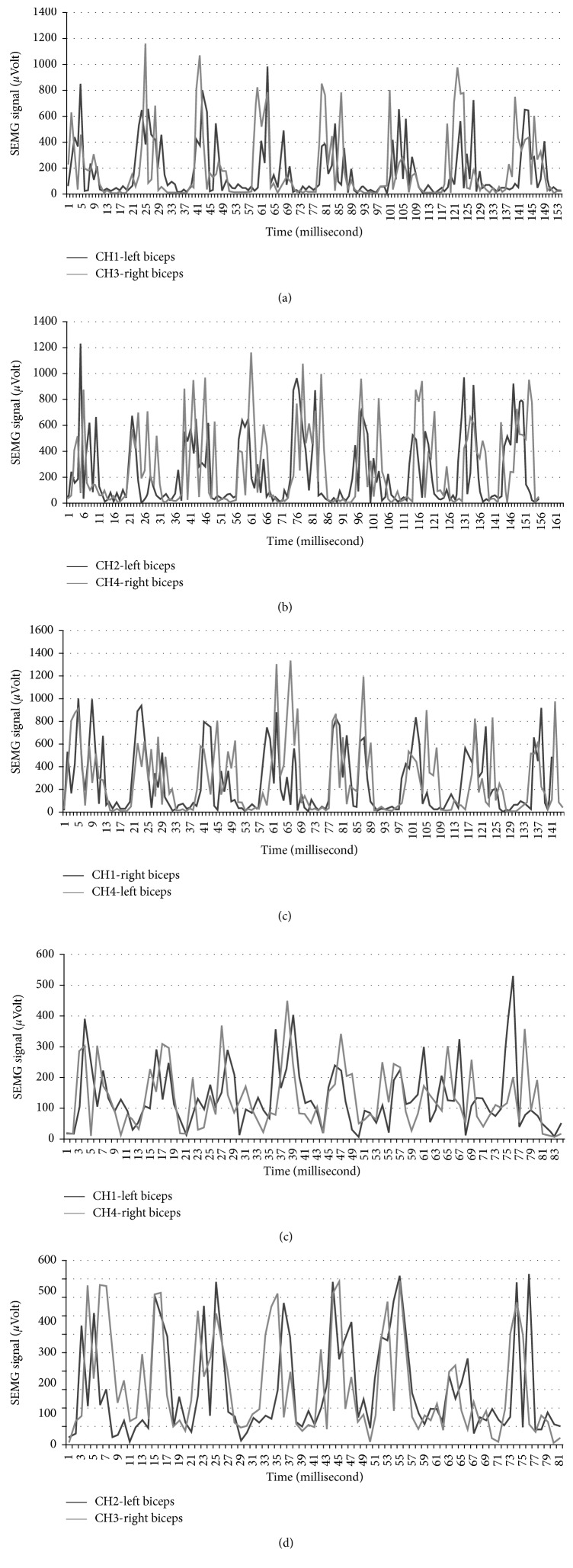
Data of other participants obtained from these trainings. (a) Results of participant number one. (b) Results of participant number three. (c) Results of participant number five. (d) Results of participant number six. (e) Results of participant number seven.

Other data of training performed by participant number four are shown in Figure 4(b). In this training, LBB data were obtained from CH3 (the third channel of the SEMG circuit) and RBB data were obtained from CH4 (the fourth channel of the SEMG circuit). When the results obtained are investigated in accordance with contraction and relaxation situations as specified in [65, 66] which consultant professor pointed, it is observed that BB muscles contract and relax normally in Rep1, Rep5, Rep7, and Rep9 and BB muscles contract fast and relax normally in Rep2. It is observed that the left BB contracts more than the right BB does and both relax normally in Rep3, that the required support is taken from other regions and movement is ruined in Rep4 and that the left BB muscle contracts more, the right BB muscle contracts normally and both relax normally in Rep6 and Rep8. It is observed that the left BB contracts normally and the right BB contracts more and both relax normally in Rep10, in which distortion in movement appears as a result of fatigue in Rep11 and Rep12. In addition, the data of other participants obtained from these trainings are presented in Figure 5.

**Figure 6:**
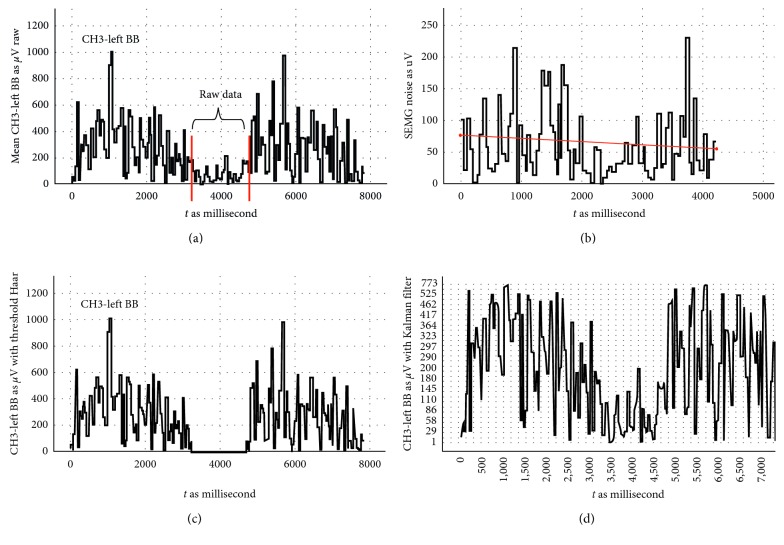
Comparison of the filtering results. (a) SEMG data without the filter. (b) Premeasurement for threshold filter. (c) SEMG signal with threshold + HW filter. (d) SEMG signal with Kalman filter.

In Figure 6, the data, processed with TCHW and Kalman filters, of two repetitions in training, belonging to the right BB muscle, conducted by the participant numbered 4, are shown. In this Figure, 6(a) shows the analogue filtered state of the SEMG signal, and 6(b) shows the preliminary measurement of the threshold cut-out. The average and standard deviation measured here were found as 61.11 ± 51.61 *μ*V, and the slope was found as 0.005⁰. The signal filtered with TCHW after this procedure is shown in 6(c), and the signal processed through Kalman filter is shown in 6(d). Filtering results indicate that the TCHW method produces better results in filtering unwanted signals and contraction detection compared to the method of Kalman filter. As a result of these processes, it was decided to use TCHW filter in our system.

### 3.2. Comparison Results with the Existing Biomedical System

**Figure 7:**
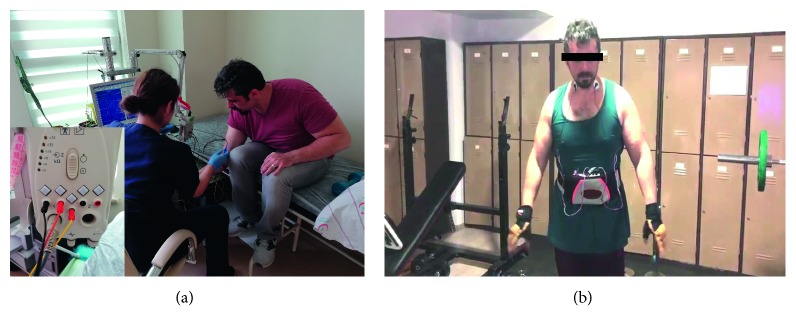
(a) A measurement taken in the hospital environment and a photograph of the current biomedical system. (b) A photograph taken at the gym before training.

The accuracy of the data obtained from our system was compared through the data belonging to two men and two women with the SEMG device in Karaman State Hospital (Figure 7).

As shown in Table 3, this procedure was carried out through the data of 108 measurements in total, obtained through volunteers being unattached, lifting dumbbells of 5 kg and the maximum weight they could lift isometrically (1 RM) first in the gym, then in the hospital system for three times with breaks of 90 seconds. In this procedure, first the data given from the hospital system were recorded and then the moving RMS was calculated on the analogue and digital filter data obtained from the system.

In the system designed as a result of this measurement, accuracies of 90.95% ± 3.35 for the left BB and 90.75% ± 3.75 for the right BB were obtained.

### 3.3. Moving RMS and %MVC Values

**Figure 8:**
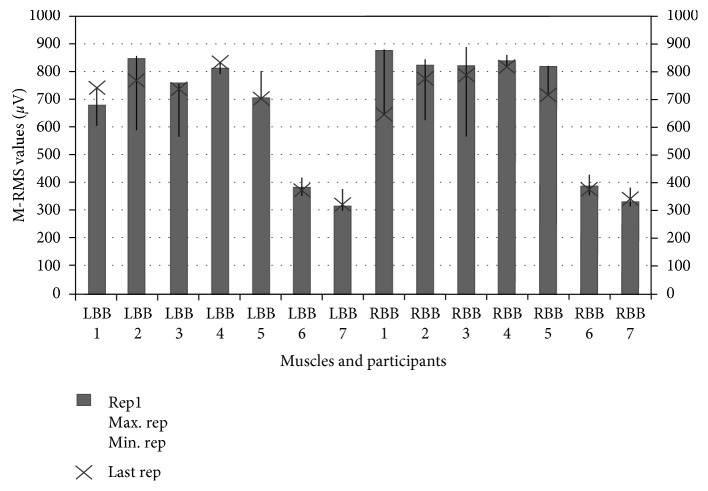
ADBC results of participants.

During the training, the volunteers were asked to perform a second training in order to obtain the moving RMS values given back to the user as feedback. The results are presented in Figure 8 and Table 4 in terms of ease of investigation.

Thus, it can be seen that the system can achieve minimum and maximum values of biopotential changes in muscles during training as in [66, 67].

Finally, the users were asked to lift 5 kg of dumbbell and the maximum weight they could lift. Thus, the %MVC was measured to be used in performance feedback through the obtained moving RMS values. The results obtained are presented in Table 5.

If Table 5 is analysed, it can be seen that the system can measure efficiency during training with the success rate of 96.87% ± 2.74 based on %MVC.

When data obtained from the designed SEMG system are compared with data obtained from the systems used in the biomedical field, it is seen that it has 90.85% accuracy. As digitally filtered data are compared, it is seen that TCHW method produces better results compared to Kalman filter. TCHW can soften data as processable and can also completely filter out unwanted signals between muscle contractions. It also eliminates the distortions in data expressed as artifact. Kalman filter appears to soften the data but not to be able to completely filter the signal between muscle contractions. Moreover, it is seen that the system can scale the strength obtained as moving RMS during the training on the basis of %MVC with the success rate of 96.87% ± 2.74 in terms of efficiency. This allows the data obtained to be used in the simultaneous performance monitoring and analysis of athletes.

## 4. Conclusion

Thanks to this system, it is thought that athletes will be able to examine their performances instantly for each training and make their training more efficient. It is possible to create intelligent training corners by using the system in gyms. It is thought that the system can easily be used by athletes, trainers, kinesiologists, and rehabilitation experts in bodybuilding trainings and rehabilitation processes. It is possible to improve system features by increasing the number of channels, further reducing the PCB size and adding extra sensor. It can be possible to follow more complicated movements (deadlift, barbell row, etc.) by increasing the number of channels. By making the size of system smaller, it can be possible to place it into textile product. In addition, by adding the pulse oximetry sensor to the system, oxygen consumption can be observed during the training. In our future studies, it is being thought of supporting the system with an image processing system in order to determine movement distortions in addition to use it for monitoring training performance and efficiency.

## Figures and Tables

**Table 1:** Comparison of the SEMG acquisition systems.

System	Signal type	Number of channels	Gain	ADC resolution (bits)	Wearable	Filter type	Contraction detection	Real-time MVC norm.	CMRR	Connection type
Proposed system	SEMG	4	4400	12	Yes	Hardware + software	Yes	Yes	>90	Bluetooth
WB-EMG	SEMG	1	100–10000	12	No	No	No	No	>90	Bluetooth
Biometrics datalog	SEMG	8	1000	14	No	No	No	No	>90	Bluetooth
Myo armband	SEMG	8	≥1000	8	Yes	Notch	No	No	>90	Bluetooth
Delsys Trignio	SEMG	16	909	16	No	Notch	No	No	>90	RF
BITalino	SEMG	Up to 6	1000	6–10	Yes	No	No	No	>90	Bluetooth
Mbody3	SEMG	Up to 6	≥1000	24	Yes	Hardware + software	No	No	>90	Bluetooth
Mpower	SEMG	4	≥1000	—	Yes	Hardware + software	No	No	>90	Bluetooth
MyoTrac	SEMG	2	≥1000	14	Yes	Butterworth	No	No	>90	Bluetooth
MyoWare	SEMG	1	≥1000	—	Yes	No	No	No	>90	Bluetooth
Shimmer	SEMG	Up to 60	≥1000	16	Yes	Hardware + software	No	No	>90	Bluetooth
*Hospital*	SEMG	8	1–10000	24	No	Hardware + software	Yes	No	>90	Usb

**Table 2:** Information about age, gender, weight, and height of the subjects.

Participant no.	Age	Gender	Weight (kg)	Height (cm)
1	21	Male	80	163
2	25	Male	82.3	178
3	29	Male	87	180
4	33	Male	85	177
5	37	Male	104.6	193
6	24	Female	70	180
7	27	Female	68	172

**Table 3:** Moving RMS Results in Gym and Hospital. Note that “M” denotes the measurement number; “BB” denotes biceps brachii; “S” denotes system; “H” denotes hospital, “MN” denotes muscle name.

Participants/weight (no./kg)														
M	MN	Type	1/idle	2/idle	3/idle	4/idle	1/5	2/5	3/5	4/5	1/25	2/25	3/15	4/12.5
I	Left BB	S	70.69	69.72	51.18	43.82	123.69	129.54	97.54	93.64	914.7	935.98	566.98	547.64
		H	67.13	72.31	47.24	45.9	137.42	141.94	108.66	101.05	950.94	1112.53	616.53	604.36
	Right BB	S	71.4	69.75	49.66	42.45	119.11	127.41	96.86	93.95	960.71	937.69	565.69	515.43
		H	69.64	70.51	50.22	43.93	135.57	143.13	107.93	97.14	943.82	1117.15	615.15	545.64

II	Left BB	S	69.86	68.84	52.03	43.15	121.82	128.9	95.71	93.8	907.35	934.5	563.5	518.06
		H	70.39	69.61	51.76	43.75	138.87	139.69	105.78	101.55	942.14	1116.89	614.89	595.59
	Right BB	S	69.84	71.34	49.01	46.68	122.96	126.95	96.5	93.61	950.6	932.61	562.61	526.48
		H	71.82	67.83	50.25	43.27	136.52	142.8	106.73	102.46	1002.4	1110.94	612.94	598.81

III	Left BB	S	69.45	70.89	50.96	46.22	124.61	128.91	97.88	89.07	907.15	934.34	564.34	511.19
		H	68.57	69.97	50.24	46.71	138.81	139.69	106.74	105.67	1000.8	1115.11	614.11	583.55
	Right BB	S	71.64	67.65	52.01	43.55	122.97	129.74	95.38	92.52	948.63	930.36	562.36	539.49
		H	68.56	69.63	49.72	44.79	135.94	145.8	107.5	104.28	1000.9	1110.61	612.61	543.35

**Table 4:** Moving RMS results in gym as training feedback.

Muscles and participants	Rep1	Rep2	Rep3	Rep4	Rep5	Rep6	Rep7	Rep8
LBB 1	862	798	738	683	782	556	715	741
LBB 2	845	779	852	786	590	812	796	766
LBB 3	757	725	721	560	712	699	645	736
LBB 4	810	841	840	804	828	832	791	830
LBB 5	704	802	651	670	604	354	558	701
LBB 6	387	413	395	354	367	403	381	370
LBB 7	316	328	372	346	377	302	328	319
RBB 1	876	833	811	790	815	846	704	653
RBB 2	823	817	847	834	649	747	621	770
RBB 3	821	793	766	696	566	884	685	785
RBB 4	832	853	856	821	819	808	809	815
RBB 5	815	763	750	753	718	707	725	714
RBB 6	389	422	418	350	371	402	361	378
RBB 7	331	380	365	351	372	348	314	341

**Table 5:** %MVC results in gym.

Muscle name	Part. no.	kg	SMVC (*μ*V)	MVC (*μ*V)	%MVC
LBB	1	5	138.3	850.26	16.26
		17.5	845.47	850.26	99.43
	2	5	138.30	992.6	13.93
		25	987.15	992.6	99.45
	3	5	152.6	960.13	15,89
		20	898.25	960.13	93,55
	4	5	155.4	963.30	16.13
		20	929.1	963.30	96.44
	5	5	158.9	967.5	16.42
		20	956.76	967.5	98.88
	6	5	89.5	615.7	14.53
		15	614.74	615.7	99.84
	7	5	93.5	545.2	17.14
		12.5	538.9	545.2	98.84

RBB	1	5	136.5	875.9	15.58
		17.5	854.15	875.9	97.51
	2	5	140.22	996.4	14.07
		25	985.92	996.4	98.94
	3	5	154.42	972.4	15.88
		20	893.56	972.4	91.89
	4	5	156.8	968.7	16.18
		20	930.76	968.7	96.08
	5	5	164.45	972.65	16.90
		20	910.5	972.65	93.61
	6	5	97.1	614.4	15.80
		15	609.9	614.4	99.26
	7	5	92.2	515.16	17.89
		12.5	506.3	515.16	98.28

## Data Availability

The data used to support the findings of this study are included within the article.
